# The challenge of disappearing colorectal liver metastasis: balancing considerations between tumor biology and clinical consequence for liver surgery

**DOI:** 10.1007/s10585-025-10357-y

**Published:** 2025-07-12

**Authors:** Daniel Ansari, Jenny Rystedt, Kjetil Søreide, Maria Lindberg, Roland Andersson

**Affiliations:** 1 Department of Surgery, Clinical Sciences Lund, Lund University, Skåne University Hospital, Lund, Sweden; 2 Department of Gastrointestinal Surgery, Stavanger University Hospital, Stavanger, Norway; 3 Department of Clinical Medicine, University of Bergen, Bergen, Norway

**Keywords:** Disappearing liver metastasis, Colorectal cancer, Definition, Diagnosis, Treatment

## Abstract

The modern use of neoadjuvant and conversion systemic therapy in patients with colorectal cancer liver metastasis (CRLM) has improved resection rates and changed the borders between “resectable” and “unresectable” disease. Also, the use of preoperative systemic therapy has resulted in an increased frequency of disappearing liver metastasis (DLM). The optimal management of DLM is still controversial. In this review, we explore the current literature and highlight key findings relating to the tumor biology, diagnosis and treatment options of DLM. The definition of DLM should be based on hepatobiliary contrast MRI, which is the most sensitive preoperative imaging method. Patients with DLM are younger and more often have normalized their CEA-levels, and they have a better survival than those without DLM, likely reflecting favorable tumor biology and effective treatment response. Recent data indicate that molecular profiling (e.g. APC mutations) may predict CRLM at highest risk for vanishing after chemotherapy. However, just because the lesion has disappeared on imaging does not mean that there is a complete histopathological response. However a “watch and wait” strategy for patients with DLM is not associated with a reduced survival compared to resected DLM, but may be associated with a higher rate of recurrence often available for “rescue therapy”, i.e. ablation or resection at the time when DLM recur and become visible. Furthermore, very few of “blind resections” of DLM contain viable tumor cells. International surveys among practicing hepatobiliary surgeons have revealed a widespread variation in the clinical management of DLM. In the future, biopsy and sequencing of metastases may be considered for therapeutic decision making in patients with CRLM considering the intricate tumor heterogeneity and clonal evolution of the disease.

## Introduction

Each year approximately 1.9 million new cases of colorectal cancer are diagnosed with 900,000 associated deaths [1]. The propensity of cancer cells to metastasize to certain organs is termed “organotropism”, which is regulated by various factors, such as blood flow pattern, cancer subtype, host organ microenvironment (referred to as the ‘metastatic niche’), and interactions with local cells [2]. For colorectal cancer, the liver is the most common site of metastasis and represents the disease burden that usually determines the longevity of patients with disseminated disease. Lung metastasis are also relatively common, but in comparison to liver metastasis, lung metastasis usually demonstrates slower growth and most commonly does not constitute the single factor that determines cancer survival. Hence, controlling the liver disease is regarded as a priority both from a systemic treatment and loco-regional management level in oncological care, with resection aimed at a radical removal (R0-resection) as the primary goal in achieving an “attempt at cure”.

Up to 50% of patients with colorectal cancer will develop liver metastasis at some point during the course of their disease [3], either at time of presentation (synchronous) or, in the course of follow-up after primary resection (metachronous) [4, 5]. Surgical resection is the main curative treatment option for colorectal liver metastasis (CRLM), but only about 20–25% of patients with CRLM present with initially resectable disease [6]. For patients with unresectable disease, systemic therapy is started with two primary aims; either as a conversion therapy attempt with the aim to convert unresectable or borderline resectable to a resectable situation, or; purely as a palliative intent. Furthermore, systemic therapy may also be given to patients with initially resectable disease in an attempt to address micrometastatic disease earlier and improve outcomes, although the data to support this is weak at best [7, 8], and with considerable variation in practice globally. For patients with synchronous CRLM most guidelines advocate neoadjuvant chemotherapy also for up-front-resectable CRLM [9]. The treatment landscape for CRLM, even among those who are resected, has become complex and heterogeneous with several pathways interchanging in care [10].

Advancements in more effective chemotherapy drugs and biological therapies have improved radiological response, tumor downstaging and survival rates [5]. Available treatment now includes chemotherapy (oxaliplatin, irinotecan), biologicals against VEGF or EGFR (bevacizumab, cetuximab) and immunotherapy for those with defect mismatch repair [11, 12]. Remarkable response has been the result from some of these combination therapies, rendering more patients resectable as a result. However, in some instances the lesions may vanish, with no radiological clear sign of disease. This may pose a challenge for the team treating the patient. This situation was first described by Elias and colleagues in the year 2004 in a series of 11 patients [13].

Complete radiological response of a CRLM following chemotherapy administration is termed “vanishing” or “disappearing” liver metastasis (DLM). However, a complete radiological response is not the same as a complete pathological response [14]. Therefore, resecting all original sites of the disease, if it can be safely performed, has traditionally been the main strategy. Such a strategy has been associated with a reduced rate of intrahepatic recurrence [15]. However, the optimal management of DLM is still a matter of controversy since pre-operative diagnostic imaging in the studies varied and there are no consensus guidelines. Furthermore, the natural history of DLM is not fully elucidated and treatment resistance may become evident as the disease progresses due to tumor heterogeneity, clonal evolution and selective pressures of therapy [16, 17].

Here we review recent studies concerning the diagnosis and management of DLM. To truly understand and effectively treat DLM, an integrative approach that takes into account tumor biology is necessary. Thus, this review starts with a general pathophysiological discussion on colorectal liver metastasis (CRLM) before highlighting emerging principles in the treatment of disappearing lesions in CRLM.

## Methods

A literature search was conducted in PubMed and Embase for studies published from database inception to March 2025. The search string used was: ((colorectal) AND (disappear* OR vanish* OR missing OR "complete response")) AND ("liver metastases" OR "hepatic metastases"). Searches were limited to English-language publications. Articles were selected by consensus among the authors based on their relevance to the central themes of the review, with priority given to primary research articles and systematic reviews. In addition, targeted supplementary searches were performed to explore the pathogenesis of colorectal liver metastases, using keywords such as “colorectal cancer”, “liver metastasis”, “pathogenesis”, “tumor biology”, “tumor microenvironment”, “invasion and migration”, “pre-metastatic niche”, and “metastatic cascade”. A second supplementary search focused on mechanisms contributing to lesion disappearance, incorporating terms such as “tumor necrosis”, “fibrosis”, “chemotherapy response”, “immune response”, “radiologic response” and “false-negative imaging”. Data from included studies were synthesized across key domains, including biological mechanisms, diagnostic and imaging criteria, treatment strategies, recurrence risk, and implications for contemporary clinical practice.

### Pathogenesis of colorectal liver metastasis

Colorectal liver metastasis (CRLM) is the cancer biology end-result of a complex multistep process that involves the spread and colonization of cancer cells from a primary tumor to the liver. During the metastatic cascade primary tumor cells harboring oncogenic driver mutations invade the basement membrane. This is followed by intravasation into blood vessels or lymphatics. Circulating tumors cells may adhere to platelets to receive protection from the shear stress exerted by the blood flow as well as recognition and destruction by natural killer cells [18]. In addition, primary tumors release molecular communicators into the circulation such as chemokines, cytokines, growth factors and exosomes which interact with bone marrow immune populations as well as directly with resident cells in the liver. The resulting interactions alter the microenvironment of the liver creating a “pre-metastatic niche” which supports future downstream colonization [19]. After traveling through the circulation, disseminated cancer cells extravasate into the liver through transendothelial migration. The extravasated tumor cells may either die, stay dormant or proliferate to form micrometastases and, later, macrometastases (i.e. lesions detectable by conventional imaging studies) [20].

The two leading models of metastatic dissemination are the linear and the parallel progression models. The linear model suggests that metastatic capacity is acquired late following the gradual accumulation of genetic alterations, with only a subset of cancer cells having the capacity to metastasize. The parallel model predicts independent progression of metastases arising from early disseminated tumor cells. It is important to distinguish between the linear and parallel models from a clinical perspective. In the linear model, there is high genomic similarity between the primary lesion and the metastases and the primary to tumor may be used as a surrogate to predict response to therapy. In the parallel model, there is genomic diversity between primary tumor and the metastases potentially requiring more aggressive therapy. According to the linear model, the primary tumor should be eradicated early to prevent metastatic dissemination, while the parallel model favors systemic therapy as the first line of treatment. However, the two models are not mutually exclusive. Previous studies highlight components of both linear and parallel metastatic progression in colorectal cancer, also within the same patients [21–23].

### Mechanisms of disappearing lesions

**Fig. 1:**
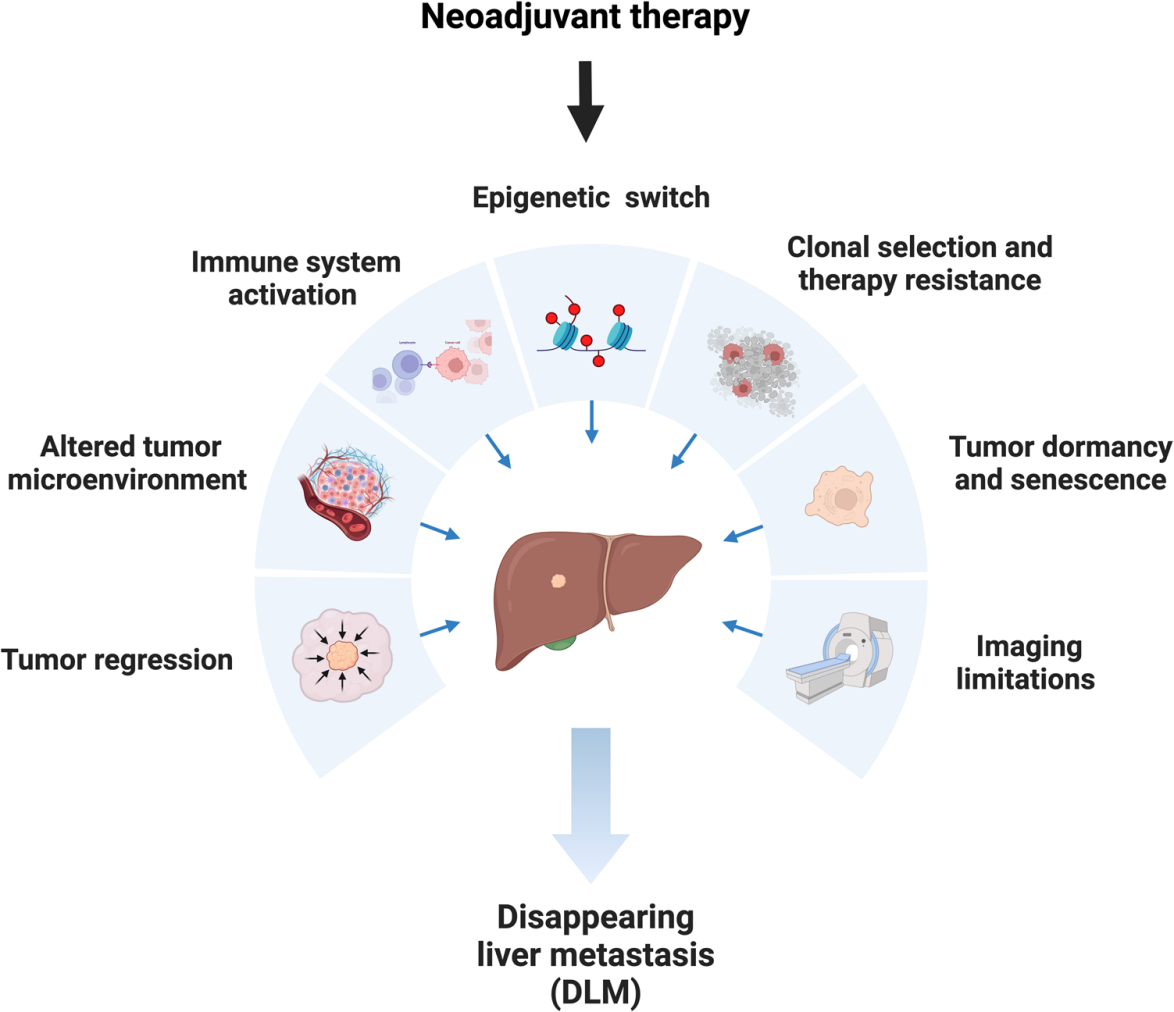
Potential mechanisms underlying the occurrence of DLM. Created with BioRender.com

With increasing efficacy of systemic cancer therapy, liver tumors can be downsized to the extent that the lesions vanish, a phenomenon termed “disappearing liver metastasis” (DLM). The survival of patients with DLM is longer than for those without DLM and, reflects the response to the systemic therapy and inherent tumor biology of the lesion [24]. Notably, DLM may occur in a lesion-by-lesion fashion, that is, a patient may well have one or more DLMs yet have other sites with visible metastatic lesions. The disappearance of CRLM following systemic therapy is influenced by several factors, as shown in Fig. 1. Systemic chemotherapy (e.g. oxaliplatin, irinotecan) can reduce tumor size by DNA damage, apoptosis, as well as chemotherapy-induced immunogenic cell death [25]. Anti-VEGF (e.g. Bevacizumab) and anti-EGFR (e.g. Cetuximab) therapies inhibit blood supply and growth signaling, leading to tumor shrinkage [26, 27]. Immunotherapy in MSI-H tumors (e.g. PD-1 inhibitors) enhances immune recognition, enabling T-cells to eliminate metastases [28]. Neoadjuvant therapy may also induce an epigenetic switch and viral mimicry, making the cancer cells difficult to distinguish from normal cells on imaging [29, 30]. Dead tumor cells are replaced by fibrotic tissue, which can mask residual disease and leading to difficulties with detection by conventional imaging [31]. Some cells may also become dormant, but remain viable, leading to later recurrence [32].

### International consensus definition of DLM

Currently, there is no accurate method to identify patients (or, lesions) that will have a remarkable response before systemic treatment is administered. However, just because the lesion has disappeared on imaging does not mean that there is a complete histopathological response. Magnetic resonance imaging (MRI) can detect some DLMs that are not visible on computed tomography (CT). Hence, MRI performed with liver-specific contrast has been considered a standard for several years, and as recently confirmed in the CAMINO study [33]. Specifically, a hepatobiliary contrast MRI (gadoxetic acid enhanced MRI) has shown high sensitivity in detecting DLM [34]. Therefore, a recent international consensus statement defined DLM as any lesion present on a baseline contrast MRI which is no longer visible on hepatobiliary contrast MRI after systemic chemotherapy [4]. A scar on cross-sectional imaging may be evidence of treatment response, but should not be termed DLM if it is detected on hepatobiliary contrast MRI. Also, reduced or normalized CEA levels have been associated with a higher likelihood for “true” pathological response in DLM.

### How common is DLM?

The reported frequency of DLM is highly variable in the literature, ranging from 7% and up to 48% (Table 1). The variation is likely explained by the differences in the use of liver-enhanced MRI, the variation in chemotherapy regimens used, as well as the heterogeneity in patients with CRLM subjected to pre-operative chemotherapy (as this varies considerably between regions). Although improved imaging may result in better lesion detection, the utilization of more effective chemotherapies (i.e. with doublet chemo-regimens and triplets based on mutations status) and broader indication for treatment of multiple metastases will most likely see an even higher incidence of DLM in the coming years. Of note, the findings of no viable cancer cells in lesions that are visible on imaging may also occur, making systemic therapy a “double jeopardy” in the sense that lesion visibility does not necessarily reflect effect on tumor biology and vice versa [35–37].

**Table 1:** Studies assessing surgical intervention or surveillance in patients with disappearing colorectal liver metastases

Author (Year)	Study type	Total number of patients	Patients with DLM	Imaging	Surgical resection		Surveillance		Patient survival	
					Resected DLM	CPR	DLM left in situ	Recurrence	Resection	No resection
Elias et al. (2004) [13]	RCS	104	11 (11%)	CT, MRI, IOUS	NR	NR	11 patients	3 (27%)	NR	NR
Benoist et al. (2006) [50]	PCS	586	38 (7%)	CT, IOUS	15	3 (20%)	31 lesions	23 (74%)	NR	NR
Elias et al. (2007) [51]	RCS	228	16 (7%)	CT, IOUS	NR	NR	16 patients	6 (38%)	NR	3-yr OS = 94% 3-yr DFS = 64%
Tanaka et al. (2009) [45]	RCS	63	23 (37%)	CT, PET-CT, IOUS	17	6 (35%)	27 lesions	11 (41%)	Median OS = 53 mo Median DFS = 22 mo	Median OS = 63 mo (p = 0.61) Median DFS = 16 mo (p = 0.73)
Auer et al. (2010) [52]	RCS	435	39 (9%)	CT, IOUS	68	44 (65%)	50 lesions	19 (38%)	NR	NR
van Vledder et al. (2010) [15]	RCS	168	40 (24%)	CT, MRI, IOUS	67	26 (39%)	17 patients	10 (59%)	1, 3 and 5-yr OS = 93%, 59% and 38% 1 and 3-yr intrahep DFS = 69% and 35% 1 and 3-year any site DFS: 60% and 23%	1, 3 and 5-yr OS = 94%, 64% and 64% (p = 0.31) 1 and 3-yr intrahep DFS = 40% and 16% (p = 0.04) 1 and 3-year any site RFS: 33% and 13% (p = 0.06)
Goere et al. (2011) [53]	RCS	523	27 (5%)	CT, IOUS	NR	NR	27 patients	9 (33%)	NR	3- and 5-year OS = 87% and 80% 3- and 5-yr DFS = 23% and 23%
Ferrero et al. (2012) [54]	RCS	292	33 (11%)	CT, MRI, IOUS	57	22 (39%)	10 lesions	6 (60%)	NR	NR
Ono et al. (2012) [55]	RCS	125	5 (4%)	CT, MRI, PET-CT	2	2 (100%)	42 lesions	8 (19%)	NR	NR
Arita et al. (2014) [56]	RCS	72	11 (15%)	CT, MRI, IOUS	25	9 (36%)	7 lesions	3 (43%)	NR	NR
Sturesson et al. (2015) [57]	RCS	179	29 (16%)	CT, MRI, IOUS	56	24 (43%)	4 lesions	1 (25%)	NR	NR
Owen et al. (2016) [46]	RCS	23	11 (48%)	CT, MRI	36	10 (28%)	41 lesions	21 (51%)	Median DFS = 483 days	Median DFS = 360 days (p = 0.49)
Kim et al. (2017) [58]	RCS	137	43 (31%)	MRI	8	8 (100%)	150 lesions	22 (15%)	NR	NR
Park et al. (2017) [59]	RCS	87	87 (NR)	CT, MRI, IOUS	168	47 (28%)	35 lesions	11 (31%)	NR	NR
Oba et al. (2018) [39]	RCS	184	59 (32%)	CT, MRI, IOUS	233	103 (44%)	42 lesions	6 (14%)	NR	NR
Tani et al. (2018) [60]	RCS	82	20 (24%)	CT, MRI, IOUS	78	24 (31%)	33 lesions	11 (33%)	NR	NR
Boraschi et al. (2023) [43]	RCS	52	15 (29%)	CT, MRI	4	3 (75%)	36 lesions	12 (33%)	NR	NR
Kuhlmann et al. (2023) [24]	RCS	158	32 (20%)	CT, MRI, PET	20	19 (95%)	70 lesions	25 (36%)	NR	NR

CPR, complete pathological response; CT, computed tomography; DFS, disease-free survival; DLM, disappearing liver metastasis; IOUS, intraoperative ultrasound; MRI, magnetic resonance imaging; NR, not reported; OS, overall survival; PCS, prospective cohort study; PET, positron emission tomography; RCS, retrospective cohort study

### Limitations of current imaging modalities

Accurately assessing DLM remains a significant challenge due to the limitations of imaging techniques. The diagnostic performance of CT, MRI with liver-specific contrast, positron emission tomography (PET), intraoperative ultrasound (IOUS) and contrast-enhanced IOUS (CE-IOUS) has been compared in evaluating DLM after chemotherapy [38]. The negative predictive value (NPV) was 0.79 for CE-IOUS, 0.73 for MRI, 0.54 for IOUS, 0.47 for CT and 0.22 for PET. These data suggest that CE-IOUS or MRI are the preferable imaging modalities for evaluation of DLM after chemotherapy. The benefit of MRI with hepatocyte-specific contrast is the higher sensitivity for detection of smaller, sub-centimeter lesions over CT alone. Findings of more extensive liver-involvement can have clinical implications and help guide decision-making preoperatively, such as indication for systemic therapy and test of biology. CE-IOUS is performed intraoperatively, but has a variable use globally. When combining a preoperative MRI with an intraoperative CE-IOUS only 16% of DLM will reappear in a median 2 year follow-up according to Oba et al. [39]. Recently, ultrasonography-based surgical navigation has been proposed as a useful method for localization and treatment of DLM [40]. It provides a live, virtual representation of the surgical scene, using a 3D model of the liver that includes tumors and critical structures.

### What are the risk factors for DLM?

Several predisposing factors have been associated with DLM including smaller lesions (< 2 cm), the presence of ≥ 3 liver metastases, synchronous CRLM disease, longer duration (and, number of cycles) of chemotherapy cycles and oxaliplatin-based agents [14]. Some of these factors may be related to the inherent aggressiveness of preoperative systemic therapy, i.e. synchronous presentation with several lesions may be subject to more aggressive preoperative systemic treatment than single/fewer lesions that are upfront resectable.

A recent study found that molecular profiling of CRLM can identify lesions at risk for disappearance after chemotherapy [41]. The risk was highest among tumors 1–2 cm in size with APC mutations after only 4 cycles of chemotherapy. Placement of a fiducial marker prior to the initiation of chemotherapy for these high-risk lesions was suggested as a method to improve localization and ensure that all lesions receive appropriate intervention [42]. However, this practice is not widely accepted nor adopted outside a few institutions.

### Treatment strategies for DLM

The management of DLM is controversial and currently no absolute criteria exist for either leaving the lesion in situ (“watch and wait”) or, opt for a resection/ablation of the area of previous lesion location (Table 2). As stated previously, a complete radiological response is not the same as a complete pathological response. Response rates may vary, and is also dependent on the definitions used for “disappearing” or small remnant lesions [43]. Among patients with resected DLM, the complete pathological response rates range from 20 to 100% (Table 1). A complete pathological response is more likely in younger patients (≤ 60 years) with a lower initial CEA, no disease on preoperative MRI or patients treated with hepatic artery infusion (HAI) chemotherapy. However, a recent meta-analysis [34] based on two studies [44, 45] found no significant difference in overall or recurrence-free survival if the resected specimen showed complete pathological response or not. When it comes to treatment of DLM, only a few retrospective studies have directly compared resection/ablation versus surveillance in terms of overall or recurrence-free survival [15, 45, 46] (Table 1). One study suggested that patients that have all original sites resected have a lower rate of intrahepatic recurrence compared with DLM left in situ, but overall survival was not different, likely due to the availability of rescue therapy [15]. In a meta-analysis [34] based on two studies [15, 46], there was no significant difference in recurrence free-survival between resected/ablated patients with DLM compared to those undergoing surveillance. A more recent study from Liverpool, utilizing several image modalities, including CT, MRI and PET, found in the per lesion analysis, recurrence after surveillance for DLM in 36%, while in 19 of 20 resected DLM no viable tumor cells were found [24]. These results are in line with a recent systematic review, which reported a lower recurrence rate for patients with resected DLM compared to those with unresected DLM [47]. The reason for adopting surveillance for DLM, when optimal diagnostic imaging has been performed, would be that we do not know which lesions that will reappear. By practicing parenchyma sparing liver surgery, not removing the site of DLMs and leaving a larger liver remnant, the patient often can be offered repeat procedures and re-resections, when metastasis reappears.

**Table 2:** Summary of recommendations from systematic reviews on the management of disappearing colorectal liver metastases

Author (year)	Aim	Number of studies	Number of patients with DLM	Recommendations
Tsilimigras et al. (2019) [14]	Evaluate imaging and management of DLM	15	479	Resection of DLM is associated with a reduced recurrence but not with improved overall survival
Araujo et al. (2020) [61]	Assess clinical decision-making for DLM	11	461	Surgery with IOUS and resection of the DLM area if residual volume is safe
Barimani et al. (2020) [62]	Investigate to what extent a DLM equates a complete response and to compare outcomes of surgery vs observation	15	491	Leaving DLM in situ may be safe if undetectable by MRI and IOUS
Muaddi et al. (2021) [38]	Examine the diagnostic performance of imaging modalities in detecting true complete response in patients with DLM	13	332	MRI or CE-IOUS are the most accurate imaging modalities. Surgical resection of visible CRLM is warranted, even if DLM remain
Nassar et al. (2022) [34]	Summarize evidence underlying different management strategies for DLM	6, including 3 systematic reviews and 3 narrative reviews	NR	The definition of DLM should be based on EOB-MRI due to the high sensitivity. Recommendations whether to resect DLM or leave in-situ cannot be established
Papakonstantinou et al. (2025) [47]	Assess recurrence in unresected DLM and complete pathological response in resected lesions	10	326	Resection of initial metastatic sites is recommended to reduce recurrence risk

CE-IOUS, contrast enhanced intraoperative ultrasound; DLM, disappearing liver metastasis; EOB-MRI, gadoxetic acid-enhanced magnetic resonance imaging; IOUS, intraoperative ultrasound; MRI, magnetic resonance imaging

### Contemporary practice among surgeons

A recent international survey among practicing hepatobiliary surgeons revealed widespread variation in the clinical management of DLM [48]. If the DLM was not identified in the operating room with intraoperative ultrasound, 48% of surgeons chose observation (“watch and wait”) and 31% would resect if the presumed area was superficial. A small proportion (13%) would resect irrespective of depth, and 7% would ablate using anatomic and vascular landmarks. Inconsistencies and disagreement in how DLM is handled is even highlighted in a more recent survey, demonstrating very poor agreement among experts for how to manage DLM [49].

## Conclusion

**Fig. 2:**
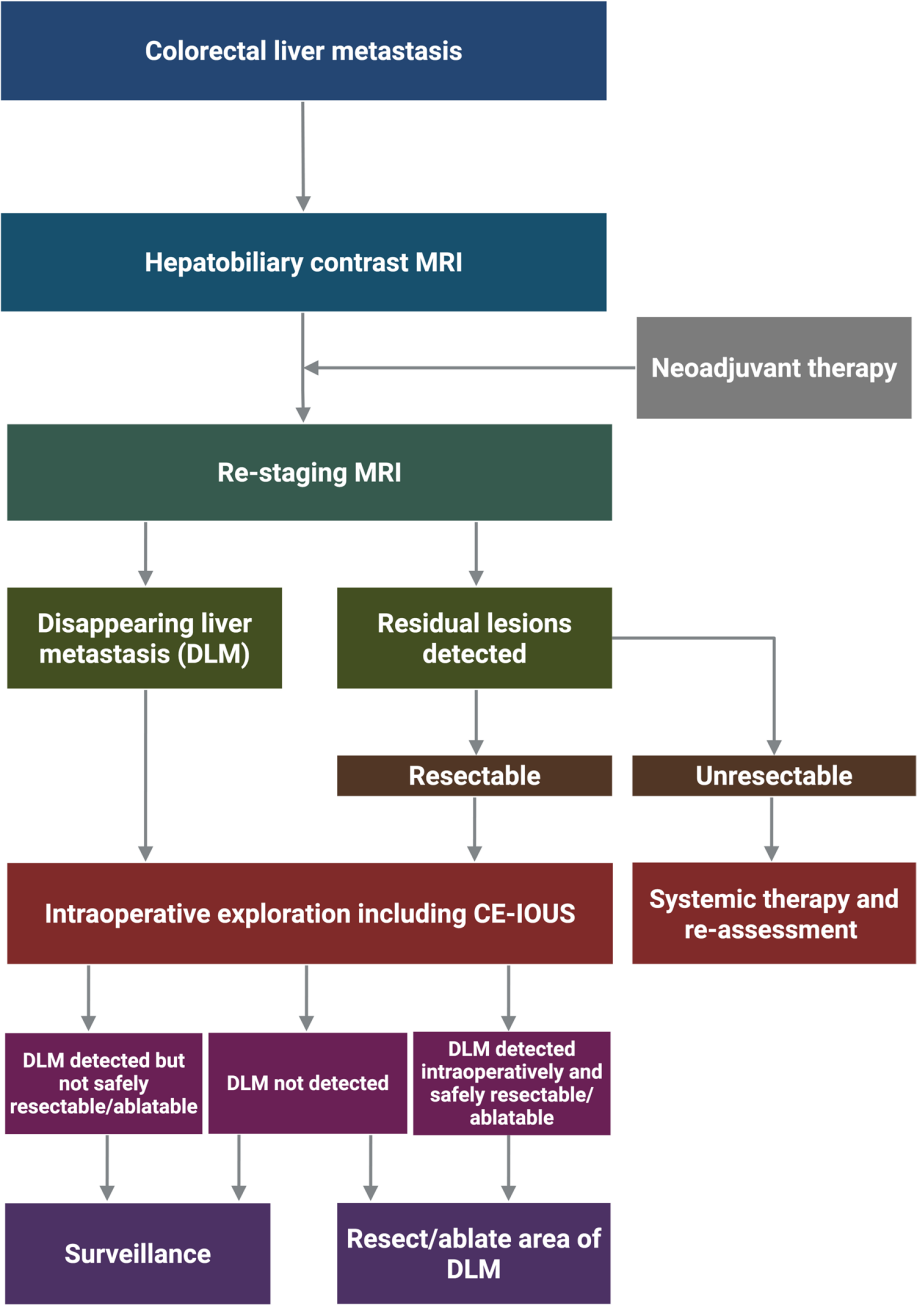
Algorithm for the management of DLM. CE-IOUS, contrast enhanced intraoperative ultrasound; DLM, disappearing liver metastasis; MRI, magnetic resonance imaging. Adapted from reference [25]. Created with BioRender.com

Modern systemic therapies have improved overall survival for patients with CRLM and increased the incidence of DLM. Although there are no consensus guidelines for the management of DLM, several recommendations can be made based on current level of evidence (Fig. 2). While there is coherent evidence for diagnosis and staging, the recommendations concerning treatment are based on incoherent evidence. Prospective, multicenter studies are ongoing to evaluate the appropriate management of DLM (e.g. LORDS-M Study), but randomized trials are still lacking in this area. In the future, as systemic therapies continue to advance, it will become increasingly crucial for imaging modalities to accurately differentiate true complete responses from residual disease, enabling more precise surgical and ablative strategies. The integration of molecular profiling to assess tumor biology and guide the intensity of local treatment, together with biomarker-driven surveillance, may support more personalized and adaptive treatment planning.

## Data Availability

No datasets were generated or analysed during the current study.
